# An optimal algorithm for mmWave 5G wireless networks based on neural network

**DOI:** 10.1016/j.heliyon.2023.e17580

**Published:** 2023-06-23

**Authors:** Liang Chen, Shebnam M. Sefat, Ki-Il Kim

**Affiliations:** a Jilin Provincial Institute of Education, Chang Chun 130022, China; b Department of Computer Science, Independent University, Bangladesh; c Islamic University Centre for Scientific Research, The Islamic University, Najaf, Iraq; d Department of Computer Science and Engineering, Chungnam National University, Daejeon 34134, South Korea

**Keywords:** mmWave communication, 5G networks, Optimization algorithm, Channel estimation

## Abstract

Fifth generation (5G) wireless networks are based on the use of spectrum blocks above 6 GHz in the millimeter wave (mmWave) range to increase throughput and reduce the overall level of interference in very busy frequency bands below 6 GHz. With the global deployment of the first commercial installations of 5G, the availability of multi-Gbps wireless connections in the mmWave frequency band becomes closer to reality and opens up some unique uses for 5G. Although, mmWave communication is expected to enable high-power radio links and broadband wireless intranet, its main challenges are inherent poor propagation conditions and high transmitter-receiver coordination requirement, which prevent it from realizing its full potential. When smart reflective surfaces are used in mmWave communication, channel state information becomes complex and imprecise. In this study, a hybrid intelligent reflecting surface consisting of a large number of passive components and a small number of RF circuits is proposed as a solution. Then, an improved deep neural network (DNN)-based technique is proposed to estimate the effective channel. The proposed technique provides better channel estimation performance according to the simulation results and improves the quality of service.

## 1 Introduction

Fifth generation (5G) communication networks will use massive multiple-input multiple-output (MIMO) technology for fading transmission [[1], [2], [3], [4], [5]]. However, a huge MIMO system consumes a lot of power, making it difficult to put into practice [[6], [7], [8], [9], [10], [11], [12]]. Moreover, this causes pilot pollution and affects the accuracy of channel estimation [[13], [14], [15], [16], [17], [18]]. Digital beamforming and full-resolution ADCs are impractical for large mmWave MIMO systems [19]. This is because the ADC power consumption increases exponentially with the number of quantization bits [20,21] and the hardware cost increases significantly [22,23]. Power consumption [24,25] and system complexity [26]. Reference [27] proposes a channel estimation method based on interference cancellation. Intelligent Reflective Surfaces (IRS) are used for auxiliary communication to further improve system efficiency. There are multiple passively reflective IRS parts that together form a plane. On the other hand, careful manipulation of amplitude and phase can change the propagation environment [28]. On the one hand, managing the propagation environment can increase the energy efficiency and spectral efficiency of wireless communication [[29], [30], [31]]. However, adding an IRS complicates the channel and introduces some difficulty in channel estimation.

A new IRS hardware structure, randomly distributed active reflective elements within passive reflective elements, and a channel estimation approach based on deep learning and compressed sensing are all proposed in Ref. [32]. On the other hand, the CS algorithm proposed in this scheme does not fully exploit the sparsity of the channel and requires a large number of active components to improve performance, resulting in high cost and complexity. On the other hand, the proposed neural network does not take phase effects into account when training the output, and the real data are all complex, resulting in poor channel estimation accuracy. References [33,34] propose a channel estimation strategy based on switching state control. In this strategy, each time his slot he turns on only one her IRS element so that the user's reflected channel is not affected by reflected signals from other channels. His IRS element channelized. However, this solution requires the amplitude of each reflective element to be controlled individually, increasing cost.

Additionally, some studies recommend cascaded channel estimation techniques. To minimize the training cost, a neural network deep denoising approach supporting CS broadband channel estimation is proposed in Ref. [35]. Reference [36] proposed a sparse matrix factorization method. The cascaded channel estimation approach used in Refs. [35,36] is practical for channel estimation, but has the following drawbacks: Distinguishing between different types of channel information can be difficult. Additionally, some studies recommend cascaded channel estimation techniques. To minimize the training cost, a neural network deep denoising approach supporting CS broadband channel estimation is proposed in Ref. [35].

In this work, a hybrid (passive/active) IRS structure is proposed and applied to mmWave communication systems. In this structure, the constrained passive components of the IRS are equipped with separate RF chains.

The main contributions of this study are as follows.

• In this work, a hybrid (passive/active) IRS structure is proposed and applied to mmWave communication systems. In this structure, the constrained passive components of the IRS are equipped with separate RF chains.
• Reduce costs by estimating mobile station (MS) and IRS channels separately. In particular, the proposed technique consists of estimates of channel gain, angle of arrival, and angle of departure. Algorithms used for traditional classification of multiple signals can only estimate the angle of arrival.
• Simultaneously estimate departure and arrival angles using an improved multiple-signal classification technique. For channel gain estimation, complex parallel DNNs have been proposed that estimate the real and imaginary parts independently to preserve phase information and include thresholding at the output to further improve the estimation accuracy.increase. The superiority of the proposed algorithm is confirmed by numerical results.

## 2 System and channel models

### 2.1 System model

**Fig. 1:**
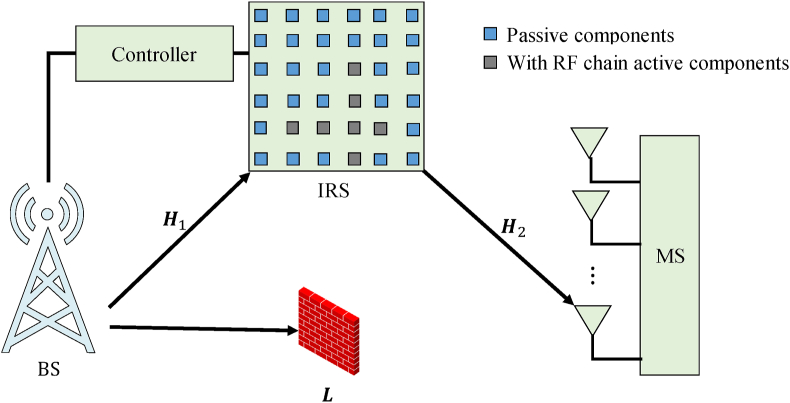
Proposed system model.

In that study, a downlink hybrid IRS structure-based mmWave communication system was proposed. The IRS consists of passively reflective NIRS components so that the phase and amplitude can be changed independently. You can also choose which NRF elements of the plus-shaped group are equipped with RF chains. IRS it works in two modes. Received and reflected states due to active NRF components [32]. In receive mode, the active component is used to estimate the channel. In reflective mode, the active element, like the passive element, simply changes the phase of the received signal before reflecting it. This allows the controller to switch the IRS behavior between these two options. Both the transmitting and receiving sides of the system contain a large number of antennas, so both BS and MS have *M* and *N* antennas. The communication system has two distribution channels. Direct connection from BS to MS and via IRS from BS to MS. These two routes have three channels.channel *L*. Transmitted directly from BS to MS. Channel ***H***1 operates from BS to IRS. Channel ***H***2 runs from IRS to MS. The mmWave transmission is supported by IRS-provided paths when an obstacle blocks the shortcut. In this study, the shortcut is assumed to be blocked by obstacles, so we use the hybrid IRS structure and mmWave communication technology. Fig. 1 shows the details of the model.

Assume that the training phase of the mmWave communication channel assisted by the hybrid IRS structure consists of *T* consecutive time frames, and each time frame contains *Q* time slots. Therefore, at the *q*th (*q* ∈ {1, …,*Q*}) time slot in the time frame *t*, the expression of the received signal at the MS is:

(1) $$y_{t,q}=H_{2}\varphi H_{1}x_{t,q}+n_{t,q}$$

where, $H_{1}\in C^{N_{IRS}\times M}$ is the channel between BS and IRS, and $H_{2}\in C^{{N\times N}_{IRS}}$ is the channel between IRS and MS; $\varphi =diag\left(\left[a_{1}e^{j\theta_{1}},a_{2}e^{j\theta_{2}},\ldots ,a_{n}e^{j\theta_{n}},\ldots ,a_{N_{IRS}}e^{j\theta_{N_{IRS}}}\right]\right)$ is phase shift, and $a_{n}\left(n\in \left[1,2,\ldots ,N_{IRS}\right]\right)$ is the magnitude; $\theta_{n}\in \left[0,2\pi \right]$ is the phase. In order to reduce the complexity of the research, this paper only studies the phase change, and the amplitude is taken as 1, that is, $a_{n}=1\left(n\in \left[1,2,\ldots ,N_{IRS}\right]\right)$; $x_{t,q}$ denotes the pilot signal; $n_{t,q}\in C^{N\times 1}$ represents the noise.

Formula (1) yields the signal received at the MS over the entire time period *t* as follows:

(2) $$Y_{t}=H_{2}\varphi H_{1}X+N_{t}$$

where, $X=\left[x_{1},x_{2},\ldots ,x_{Q}\right]\in C^{M\times Q}$ denotes the pilot signal over t; $N_{t}=\left[n_{t,1},n_{t,2},\ldots ,n_{t,Q}\right]\in C^{N\times Q}$ is the additive white Gaussian noise matrix.

At this point, the received signal at the element equipped with the RF chain on the IRS can be written as in Eq. (3):

$${\bar{H}}_{1}=GH_{1}$$

(3) $$Y_{t}^{IRS}={\bar{H}}_{1}X+N_{t}^{IRS}$$

where, G is a selection matrix of $N_{RF}\times N_{IRS}$, which is used to select the active components on the IRS; ${\bar{H}}_{1}\in C^{N_{RF}\times M}$ is the channel from BS to NRF active elements on IRS; $N_{t}^{IRS}\in C^{N_{RF}\times Q}$ is an additive white Gaussian noise matrix.

### 2.2 Channel model

Since mm-wave channels have a finite number of scattering paths and an abundance of geometric channel models, each scattering path should be consistent with the channel characteristics. The paths between the IRS and MS are expressed as:

(4) $$H_{1}=\sqrt{\frac{MN_{IRS}}{L_{1}}}\sum_{l_{1}=1}^{L_{1}}\alpha_{l_{1}}a_{IRS}\left(\gamma_{l_{1}},\varphi_{l_{1}}\right)a_{BS}^{H}\left(\theta_{l_{1}}\right)$$

(5) $$H_{2}=\sqrt{\frac{NN_{IRS}}{L_{2}}}\sum_{l_{2}=1}^{L_{2}}\beta_{l_{2}}b_{MS}\left(\theta_{l_{1}}^{\prime }\right)b_{IRS}^{H}\left(\gamma_{l_{2}}^{\prime },\varphi_{l_{2}}^{\prime }\right)$$

where, $L_{1}$ and $L_{2}$ denote the numbers of scattering paths on $H_{1}$ and $H_{2}$; $\alpha_{l_{1}}∼CN\left(0,\sigma_{l_{1}}^{2}\right)$ and $\beta_{l_{2}}∼CN\left(0,\sigma_{l_{2}}^{2}\right)$ are the gain of paths $l_{1}$ and $l_{2}$. The IRS's usage of a uniform linear array (ULA) structure in some earlier works defies reality in the name of simplicity. Therefore, the BS and MS adopt a ULA structure, and this article installs a uniform planner array (UPA). The $H_{1}$ channel response vectors are written as:

(6) $$a_{IRS}\left(\gamma_{l_{1}},\varphi_{l_{1}}\right)=\frac{1}{\sqrt{N_{IRS}}}{\left[1,\ldots ,e^{j\frac{2\pi }{\lambda }d\left(m\;\sin\left(\gamma_{l_{1}}\right)\sin\left(\varphi_{l_{1}}\right)+n\;\cos\left(\varphi_{l_{1}}\right)\right)},\ldots ,e^{j\frac{2\pi }{\lambda }d\left(\sqrt{N_{IRS}-1}\sin\left(\gamma_{l_{1}}\right)\sin\left(\varphi_{l_{1}}\right)+\sqrt{N_{IRS}-1}\cos\left(\varphi_{l_{1}}\right)\right)}\right]}^{T}$$

(7) $$a_{BS}\left(\theta_{l_{1}}\right)=\frac{1}{\sqrt{M}}{\left[1,\ldots ,e^{j\frac{2\pi }{\lambda }dm\;\sin\left(\theta_{l_{1}}\right)},\ldots ,e^{j\frac{2\pi }{\lambda }d\left(M-1\right)\sin\left(\theta_{l_{1}}\right)}\right]}^{T}$$

λ denotes the wavelength; d is the gap between array elements, which is λ/2; $\gamma_{l_{1}}$, $\varphi_{l_{1}}$ shows the angles of the azimuth and elevation plans at the IRS on the $l_{1}$ path, respectively, and $\theta_{l_{1}}$ is the departure angle at the BS on the $l_{1}$ path.

The following modifications might be made to the construction to make it more compact:

(8) $$\alpha =\sqrt{\frac{MN_{IRS}}{L_{1}}}\left[\alpha_{1},\alpha_{2},\ldots ,\alpha_{L_{1}}\right]$$

(9) $$A_{BS}=\left[a_{BS}\left(\theta_{1}\right),a_{BS}\left(\theta_{2}\right),\ldots ,a_{BS}\left(\theta_{L_{1}}\right)\right]$$

(10) $$A_{IRS}=\left[a_{IRS}\left(\gamma_{1},\varphi_{1}\right),a_{IRS}\left(\gamma_{2},\varphi_{2}\right),\ldots ,a_{IRS}\left(\gamma_{L_{1}},\varphi_{L_{1}}\right)\right]$$

More advanced and optimized in this context refers to more compact design. It is based on parameter optimization, in other words.

Through formulas (8) ∼ (10), equation (4) can be written as:

(11) $$H_{1}=A_{IRS}diag\left(\alpha \right)A_{BS}^{H}$$

Channel $H_{2}$ and Channel $H_{1}$ are analogous from an analytical standpoint, and the formulas for Channels in the same system may be approximated using mathematical equalities. For instance, two channels using the same system have a number of identical traits yet operate at a different frequency. So formula (5) can be rewritten as Eq. (12) ∼ (15):

(12) $$H_{2}=B_{MS}diag\left(\beta \right)\;B_{IRS}^{H}$$

where,

(13) $$\beta =\sqrt{\frac{MN_{IRS}}{L_{2}}}\left[\beta_{1},\beta_{2},\ldots ,\beta_{L_{2}}\right]$$

(14) $$B_{MS}=\left[b_{MS}\left(\theta_{1}^{\prime }\right),b_{MS}\left(\theta_{2}^{\prime }\right),\ldots ,b_{MS}\left(\theta_{L_{2}}^{\prime }\right)\right]$$

(15) $$B_{IRS}=\left[b_{IRS}\left(\gamma_{1}^{\prime },\varphi_{1}^{\prime }\right),b_{IRS}\left(\gamma_{2}^{\prime },\varphi_{2}^{\prime }\right),\ldots ,b_{IRS}\left(\gamma_{L_{2}}^{\prime },\varphi_{L_{2}}^{\prime }\right)\right]$$

## 3 Channel estimation

The hybrid IRS structure mentioned above aids in channel estimate on the mmWave communication system model. Since the IRS has active components, it is simple to estimate the channel ${\bar{H}}_{1}$ in receive mode between the IRS's active components and the BS. In this article, the channel ${\bar{H}}_{1}$ is estimated using the least squares (LS) approach. Its estimated expression is in Eq. (16):

(16) $${\bar{H}}_{1}=Y_{t}^{IRS}X^{H}{\left(XX^{H}\right)}^{-1}$$

By studying formula (11), it can be found that channel $H_{1}$ contains information such as array response $A_{IRS}$, $A_{BS}$, and path gain α, which means that the above information needs to be estimated before channel $H_{1}$ can be estimated.

### 3.1 Angle-of-departure/arrival Estimation for H_1_ channels

Only the angle-of-arrival (AoA) may be estimated via the multi-signal classification (MUSIC) technique traditionally used [37]. However, because the IRS uses a uniform planner array (UPA) structure, the AoA actually includes two types of information, azimuth and elevation angle, that is, the array response of the L1 path at the IRS in $A_{IRS}$. On the one hand, the angle-of-departure (AoD) must also be estimated.

The proposed augmented MUSIC method converts 3D to 2D while estimating AoA, preventing 3D estimation. The proposed method uses 3D array geometries to process real-domain data, which greatly reduces the computational cost. Furthermore, the 3D function is reduced to his 2D function parameters by extrapolation from the polarization parameter calculation. As a result, the number of iterations required to find spectral peaks is greatly reduced, as simultaneous searches for AoD and polarization parameters are avoided. In addition, the text also makes the following claims: Horizontal and vertical uniform linear arrays can be used to estimate azimuth and elevation respectively, based on the fact that active components with RF chains are placed on the IRS with a plus sign. Currently, just two types of information in each direction need to be estimated. Here it is assumed that *N*_RF_ is an odd number, $n_{x}$ denotes the number of horizontal array elements horizontal, and the number of elements on the vertical array is $n_{y}=N_{RF}-n_{x}+1$. Therefore, the response vectors of the IRS array in the horizontal and vertical directions can be obtained from Eq. (17) ∼ (18):

(17) $$a_{IRS}^{x}\left(\gamma_{l_{1}}\right)=\frac{1}{\sqrt{n_{x}}}{\left[1,e^{j\frac{2\pi }{\lambda }d\;\sin\left(\gamma_{l_{1}}\right)},\ldots ,e^{j\frac{2\pi }{\lambda }d\left(n_{x}-1\right)\;\sin\left(\gamma_{l_{1}}\right)}\right]}^{T}$$

(18) $$a_{IRS}^{y}\left(\varphi_{l_{1}}\right)=\frac{1}{\sqrt{n_{y}}}{\left[1,e^{j\frac{2\pi }{\lambda }d\;\cos\left(\varphi_{l_{1}}\right)},\ldots ,e^{j\frac{2\pi }{\lambda }d\left(n_{y}-1\right)\;\cos\left(\varphi_{l_{1}}\right)}\right]}^{T}$$

The received signal at the IRS's uniform linear array of $n_{x}$ active elements set up using RF chains is shown in Eq. (19) in the horizontal direction:

(19) $$Y_{t,x}^{IRS}=A_{IRS}^{x}diag\left(\alpha \right)A_{BS}^{H}X+N_{t,x}^{IRS}$$

$A_{IRS}^{x}=\left[a_{IRS}^{x}\left(\gamma_{1}\right),\ldots ,a_{IRS}^{x}\left(\gamma_{l_{1}}\right),\ldots ,a_{IRS}^{x}\left(\gamma_{L_{1}}\right)\right]$. At this time, the traditional MUSIC algorithm can only estimate the angle of arrival $\gamma_{l_{1}}$, but cannot estimate the angle of departure $\theta_{l_{1}}$, while the improved MUSIC algorithm can make it possible to estimate the AoA and AoD of each path through spatial spectrum analysis. In order to construct a suitable direction matrix, it is necessary to perform a vectorization operation on formula (19), and its expression is formulated by Eq. (20):

(20) $$y_{x}=vec\left(Y_{t,x}^{IRS}\right)=\underset{W}{\underbrace{\left[\left(A_{BS}^{H}X\right)⊗A_{IRS}^{x}\right]}}vec\left(diag\left(\alpha \right)\right)+vec\left(N_{t,x}^{IRS}\right)$$

where, $W\in C^{Qn_{x}\times L_{1}^{2}}$ is the direction matrix, including the AoA and AoD information. Then, determine the covariance matrix and perform eigenvalue decomposition, it is expressed in Eq. (21):

(21) $$R=E\left(y_{x}y_{x}^{H}\right)=U\Lambda U^{H}=\left[U_{s}\;U_{n}\right]\left[\begin{array}{c} \Lambda_{s}\\ 0 \end{array}\;\begin{array}{c} 0\\ \Lambda_{s} \end{array}\right]\left[\begin{array}{c} U_{s}^{H}\\ U_{n}^{H} \end{array}\right]=U_{s}\Lambda_{s}U_{s}^{H}+U_{n}\Lambda_{n}U_{n}^{H}$$

Where, U is an eigenvalue vector that can be expanded into the signal subspace $U_{s}$ and the noise space $U_{n}$ [38,39], which are respectively composed of $L_{1}$ largest eigenvalues $\Lambda_{n}$ and ${Qn}_{x}-L_{1}$ eigenvectors corresponding to small eigenvalues $\Lambda_{n}$. Finally, the direction spectrum function is obtained as in Eq. (22):

(22) $$P\left(\theta ,\gamma \right)=\frac{1}{{\left({\left[a_{BS}^{H}\left(\theta \right)X\right]}^{T}⊗a_{IRS}^{x}\left(\gamma \right)\right)}^{H}U_{n}U_{n}^{H}\left({\left[a_{BS}^{H}\left(\theta \right)X\right]}^{T}⊗a_{IRS}^{x}\left(\gamma \right)\right)}$$

By searching the $L_{1}$ poles in the direction spectrum function, the θ and γ on the $L_{1}$ paths can be estimated. The AoA estimation in the vertical direction is similar to the estimation in the horizontal direction because of 2D structure, and the improved MUSIC algorithm can also be used to estimate the elevation angle *φ* on the $L_{1}$ path []. Finally, the estimated values ${\hat{A}}_{BS}$ and ${\hat{A}}_{IRS}$ of $A_{BS}$ and $A_{IRS}$ can be obtained.

### 3.2 Path gain Estimation for H_1_ channel

**Fig. 2:**
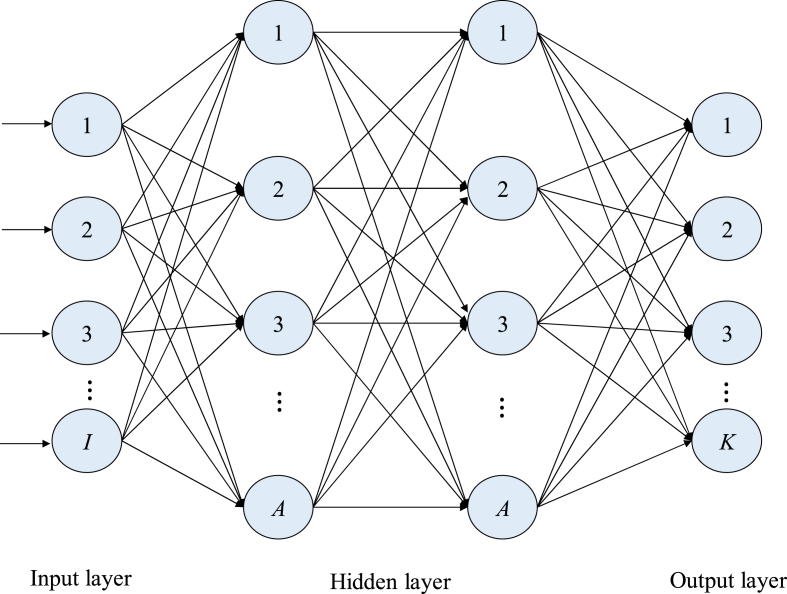
Architecture of DNN

Path gain α is important information in channel $H_{1}$, which can be estimated through channel ${\bar{H}}_{1}$. It is expressed in Eq. (23):

(23) $${\bar{H}}_{1}=G{\hat{A}}_{IRS}diag\left(\alpha \right){\hat{A}}_{BS}^{H}+N_{1}$$

where, diag(α) is the function to estimate the path gain matrix, ${\hat{A}}_{BS}$ and ${\hat{A}}_{IRS}$ are the estimated array response values of $L_{1}$ paths at BS and IRS respectively, and $N_{1}\in C^{N_{RF}\times M}$ is the AWGN matrix. In order to facilitate the following estimation, it is necessary to vectorize the channel ${\bar{H}}_{1}$ here, and its expression is:

(24) $${\bar{h}}_{1}=vec\left({\bar{H}}_{1}\right)={\left({\hat{A}}_{BS}^{H}\right)}^{T}⊗\left({G\hat{A}}_{IRS}\right)vec\left(diag\left(\alpha \right)\right)+vec\left(N_{1}\right)={\left({\hat{A}}_{BS}^{H}\right)}^{T}⊗\left({G\hat{A}}_{IRS}\right)D+vec\left(N_{1}\right)$$

where, ${\bar{h}}_{1}\in C^{MN_{RF}\times 1}$, $D=vec\left(diag\left(\alpha \right)\right)\in C^{L_{1}L_{1}\times 1}$, $vec\left(N_{1}\right)\in C^{{MN}_{RF}\times 1}$ is the additive white Gaussian noise vector. This work suggests a method based on DNN to estimate the route gain in accordance with formula (24). Although the LS method requires the determination of the pseudo-inverse function, this method requires less prior knowledge of the estimation than the LS method because only the channel information between the active components needs to be captured. Unless the matrix is perfectly ranked, it will not be possible to find a unique solution and eventually the estimation results will be in error. Adding more network layers to a DNN can improve training results, but it also increases the problem of overfitting and training complexity. The number of DNN layers is changed to balance these two criteria. Finally, with four network layers, a balance between complexity and training effectiveness is achieved by adjusting the neural network weights and learning rate to reduce the loss. The DNN contains four levels, as illustrated in Fig. 2. The input layer has *I* number of neuran, the two hidden layers have *A* number of neurons, and the output layer has K number of nuerons, and the layers making up a completely linked structure. Therefore, the weight matrix between layers of this four-layer neural network is $W_{1}\in R^{A\times 1}$, $W_{2}\in R^{A\times A}$ and $W_{3}\in R^{K\times A}$, the bias vectors are $b_{1}\in R^{A}$, $b_{2}\in R^{A}$ and $b_{3}\in R^{K}$. A nonlinear hyperbolic tangent activation function is used at the two hidden layers $\left(\tanh\left(x\right)=e^{x}-e^{-x}/e^{x}+e^{-x}\right)$ to improve the neural network performance.

According to the actual situation, the processed data consist of complex numbers. Complex phase information is lost in some previous neural network approaches that simply extract the real and imaginary parts and input them as real numbers to the neural network. As a result, the final output is only real numbers. In this work, to address this problem, we provide a method to separately estimate the real and imaginary parts using different parallel DNN architectures. We can get: It is based on the properties of neural networks and complex numerical operations.

(25) $$\left[\begin{array}{c} Re\left(\hat{D}\right)\\ Im\left(\hat{D}\right) \end{array}\right]=\left[\begin{array}{c} Re\left(W,b\right)\\ Im\left(W,b\right) \end{array}\;\begin{array}{c} -Im\left(W,b\right)\\ Re\left(W,b\right) \end{array}\right]\left[\begin{array}{c} Re\left({\bar{h}}_{1}\right)\\ Im\left({\bar{h}}_{1}\right) \end{array}\right]$$

**Fig. 3:**
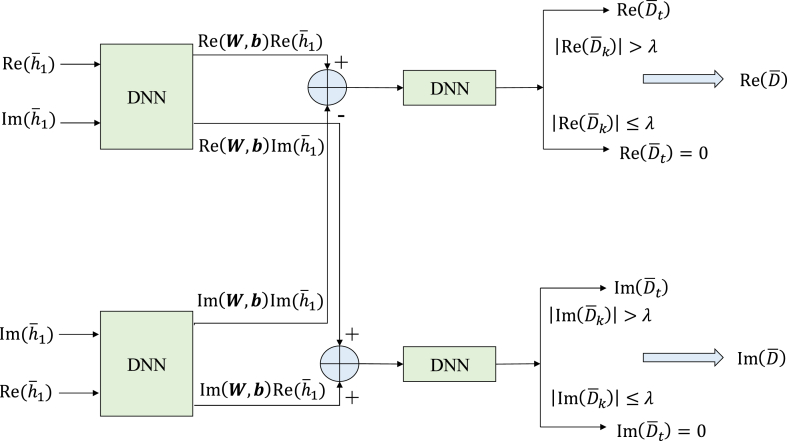
Proposed complex parallel DNN framework.

According to formula (25), the complex parallel neural network proposed in this paper is shown in Fig. 3.

The ADAM algorithm is deployed to train the neural network [40]. The goal is to reduce the loss function of the path gain matrix D. The real part's loss function and the imaginary part's loss function are, respectively:

(26) $$Loss\left(Re\left(\hat{D}\right)\right)=\frac{1}{PK}\sum_{p=1}^{P}{\left\|Re{\left(\hat{D}\right)}^{\left(p\right)}-Re{\left(D\right)}^{\left(p\right)}\right\|}_{F}^{2}$$

(27) $$Loss\left(Im\left(\hat{D}\right)\right)=\frac{1}{PK}\sum_{p=1}^{P}{\left\|Im{\left(\hat{D}\right)}^{\left(p\right)}-Im{\left(D\right)}^{\left(p\right)}\right\|}_{F}^{2}$$

where, P denotes the quantity of training samples, $Re{\left(D\right)}^{\left(p\right)}$ and $Im{\left(D\right)}^{\left(p\right)}$ represent the real and imaginary parts of the p th sample. All that is required for the proposed neural system is to determine the channel information between the active components on the BS and the IRS, input the real and imaginary parts separately to the input layer, and combine them according to Eq. (25). Communication network. Determine the values of the output layer neurons based on the loss functions given in equations (26), (27) and compute the adjusted vector. Because the channel gains matrix D contains some 0 elements, it is difficult for the trained neural network to estimate the 0s accurately, and only small values can be estimated. To further reduce the error, a threshold decision is added to the final output of the real and imaginary part estimation, and the threshold decision is given by equation (28):

$$Re\left({\hat{D}}_{k}\right)=\left\{ \begin{array}{c} Re\left({\hat{D}}_{k}\right),\left|Re\left({\hat{D}}_{k}\right)\right|>\lambda \\ 0,\left|Re\left({\hat{D}}_{k}\right)\right|\leq \lambda \\ k=1,2,\ldots ,K \end{array}\right.$$

(28) $$Im\left({\hat{D}}_{k}\right)=\left\{ \begin{array}{c} Im\left({\hat{D}}_{k}\right),\left|Im\left({\hat{D}}_{k}\right)\right|>\gamma \\ 0,\left|Im\left({\hat{D}}_{k}\right)\right|\leq \gamma \\ k=1,2,\ldots ,K \end{array}\right.$$

where, $Re\left({\hat{D}}_{k}\right)$, $Im\left({\hat{D}}_{k}\right)$ are the *k*th elements of the output vectors $Re\left(\hat{D}\right)$, $Im\left(\hat{D}\right)$ respectively; λ and γ are the judgment thresholds, both of which are 0.001 in this paper which are suitable values by which the system gives optimal performance [41,42].

### 3.3 H_2_ channel estimation

According to the above scheme, the array response estimation matrix ${\hat{A}}_{BS}\left({\hat{A}}_{IRS}\right)$ and the path gain estimation matrix $\hat{D}$ of $L_{1}$ paths at BS (IRS) in $H_{1}$ can be determined, and then the estimated value ${\bar{H}}_{1}$ of channel $H_{1}$ can be obtained [43]. In the reception mode of the hybrid IRS structure, the channel $H_{1}$ is estimated, and then in the reflection mode, the MS only needs to estimate the channel $H_{2}$ [44]. At this time, formula (2) can be rewritten as in Eq. (29):

(29) $${\bar{Y}}_{t}=H_{2}\Phi_{RF}Y_{t}^{IRS}+N_{t}=B_{MS}diag\left(\beta \right)B_{IRS}^{H}\Phi_{RF}Y_{t}^{IRS}+{\bar{N}}_{t}$$

${\bar{Y}}_{t}\in C^{N\times Q}$ is the received signal at the MS, which is reflected from an element with an RF chain on 46the IRS. For channel $H_{2}$, the above method can also be used. The ULA equipped with the horizontal direction of the RF chain and the uniform linear array furnished with the vertical direction of the RF chain still estimate the angle of departure independently [45]. Estimating the AoA and AoD from the horizontal and vertical directions using the enhanced MUSIC algorithm. Finally, the estimated value ${\bar{B}}_{MS}$ of the array response $B_{MS}$ and the estimated value ${\bar{B}}_{IRS}$ of the array response $B_{IRS}$ can be obtained. The proposed complex parallel DNN is used to estimate the channel gain. Before estimating the channel gain, the received signal ${\bar{Y}}_{t}$ is vectorized [46]. It is expressed in Eq. (30):

$${\bar{y}}_{t}=vec\left({\bar{Y}}_{t}\right)=\left[{\left(B_{IRS}^{H}\Phi_{RF}Y_{t}^{IRS}\right)}^{T}⊗B_{MS}\right]vec\left[diag\left(\beta \right)\right]+vec\left({\bar{N}}_{t}\right)$$

(30) $$=\left[{\left(B_{IRS}^{H}\Phi_{RF}Y_{t}^{IRS}\right)}^{T}⊗B_{MS}\right]\bar{D}+vec\left({\bar{N}}_{t}\right)$$

where, ${\bar{y}}_{t}\in C^{NQ\times 1}$, $\bar{D}=vec\left(diag\left(\beta \right)\right)\in C^{L_{2}L_{2}\times 1}$, and $vec\left({\bar{N}}_{t}\right)$ is an additive white Gaussian noise vector. The input of the complex parallel DNN is Re ($\bar{y}$) and Im ($\bar{y}$), after the training of the neural network, the Re ($\bar{D}$) and Im ($\bar{D}$) are estimated. Combining the estimated matrix ${\bar{B}}_{MS}$ and ${\bar{B}}_{IRS}$ of the above array response, the estimated value ${\bar{H}}_{2}$ of the channel $H_{2}$ can be finally obtained.

## 4 Simulation results

The performance of the suggested algorithm is tested, and the accuracy, using the normalized mean square error (NMSE). The analytical expression is formulated in Eq. (31) as follows:

(31) $$NMSE\left(\bar{H}\right)=\frac{1}{R}\sum_{r=1}^{R}\frac{{\left\|H_{r}-{\bar{H}}_{r}\right\|}_{F}^{2}}{{\left\|H_{r}\right\|}_{F}^{2}}$$

where R represents the number of iterations, and ${\bar{H}}_{r}$ is estimated the r th channel. The simulation in this paper assumes $Q=20,T=50,M=50,N=4,L_{1}=L_{2}=3,N_{RF}=5\;\left[48\right],R=5000$, $N_{IRS}\in \left\{16,25,36,49\right\}\text{.}$ These are considered values for the proposed system scenario. We tested the performance by setting such parameters. It can be changed to other configuration depending on the requirements.

**Fig. 4:**
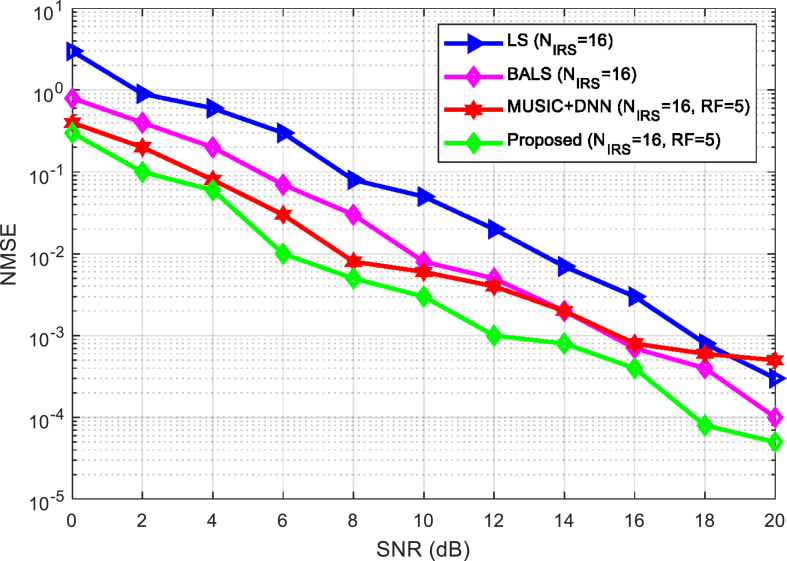
Comparison of channel estimation parameter of the algorithms under varying SNR.

When $N_{IRS}=16$, Fig. 4 compares the channel estimation algorithms (MUSIC + DNN), BALS, LS, and the suggested approach. Fig. 4 demonstrates that the suggested approach performs better than BALS and LS algorithms. Fig. 4 compares the standard DNN and the plan using the updated MUSIC algorithm. By comparison, we find that the proposed complex parallel DNN considering the phase factor can actually improve the accuracy of channel estimation.

**Fig. 5:**
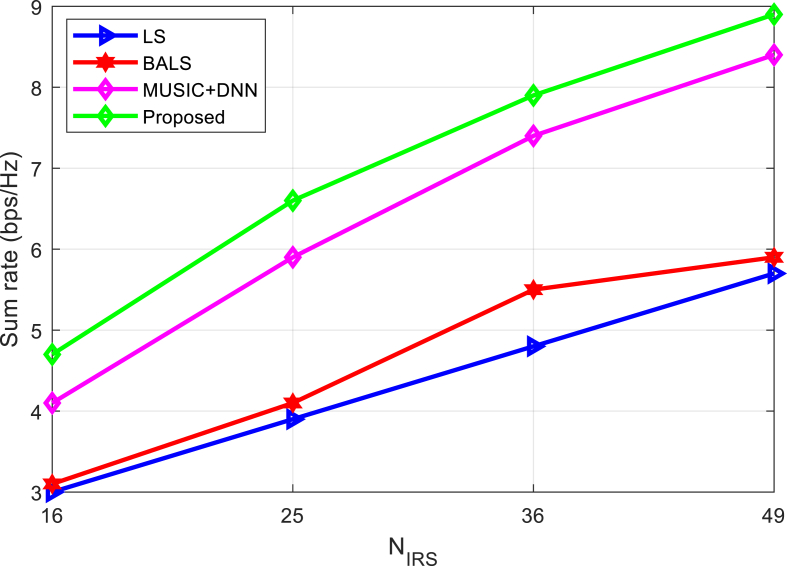
Sum rate comparison of algorithms under increasing IRF elements.

The achievable sum rate is compared in Fig. 5 with an increased number of reflecting surfaces. Its effectiveness is demonstrated by Fig. 5, which shows that the proposed method outperforms the current algorithm at any *N*_IRS_ value.

**Fig. 6:**
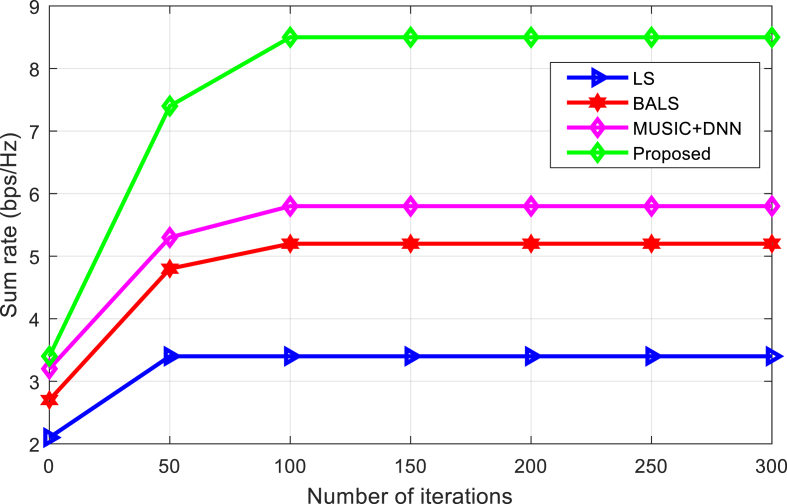
Convergence comparison of the algorithms under increasing number of iterations.

Fig. 6 evaluates the convergence of the algorithms over a series of iterations. As can be seen from Fig. 6, the proposed algorithm is fast in comparison with current techniques and approaches the target accumulation rate.

**Fig. 7:**
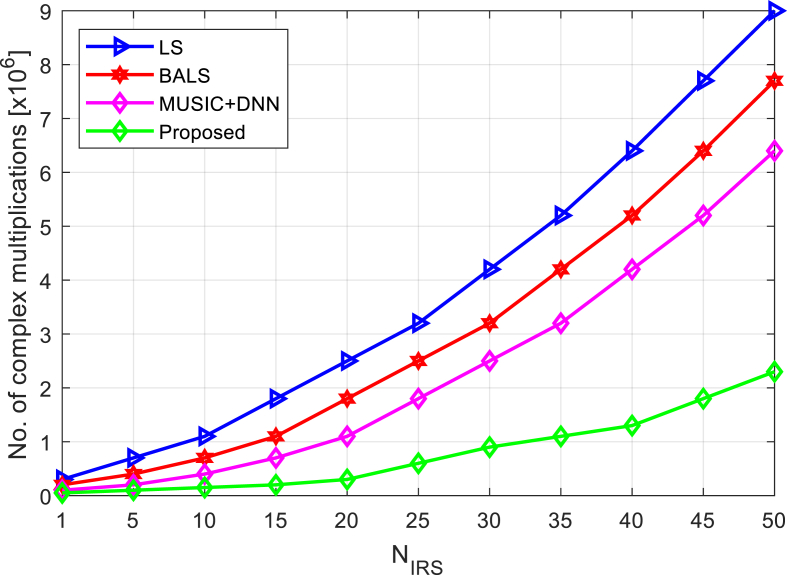
Complexity comparison of algorithms under increasing number of IRS elements.

The complexity of the algorithms is compared in Fig. 7 when there are many IRS entries. Fig. 7 shows that for all methods the number of complex multiplications increases with increasing IRS elements. However, compared to previous techniques, the proposed approach requires less complex multiplications, making it more computationally efficient and practical to implement.

**Fig. 8:**
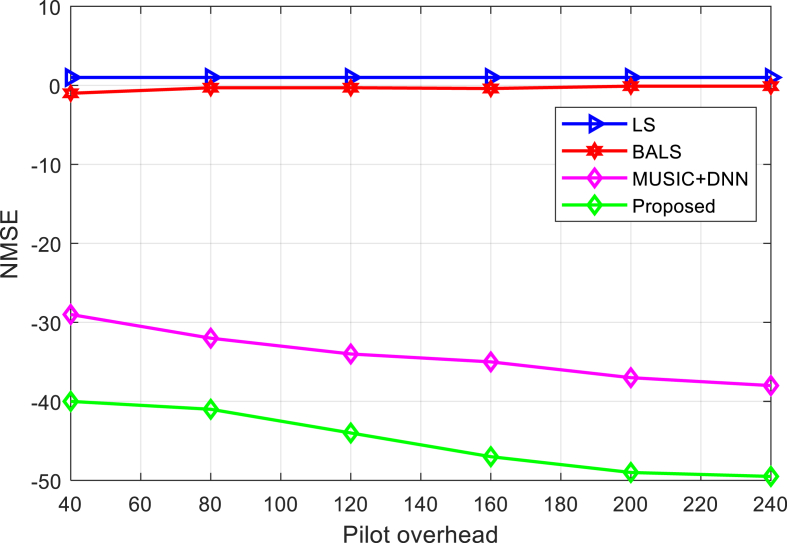
NMSE comparison of the proposed and other algorithms with different pilot overhead.

Fig. 8 compares the algorithm's NMSE for various pilot overheads. As shown in Fig. 8, the NMSE of the LS and BALS algorithms remains essentially constant over the range of pilot overhead levels. Additionally, the increased pilot overhead reduces the NMSE of the MUSIC + DNN algorithm. The proposed method has lower NMSE than the current algorithm, so it is better and feasible.

## 5 Conclusion

This article proposes a completely new channel estimation technique for millimeter-wave communication networks. By providing the IRS with a constrained RF chain, the main goal is to estimate departure angles, arrival angles, and channel gains. The proposed method aims to estimate both the AoA and AoD, since the conventional MUSIC algorithm could only estimate the arrival angle. Channel gains are then estimated using a complex parallel DNN, and the network topology can avoid phase loss during complex training. We demonstrate the feasibility of the proposed system through simulations and show its superiority by comparison with other algorithms.

## Author contribution statement

Liang Chen: Conceived and designed the experiments; Wrote the paper.

Shebnam M. Sefat: Experimentations; Analyzed and interpreted data; provided reagents, materials, analytical tools or data.

Ki-Il Kim: Contributed reagents; Analyzed and interpreted the data; Wrote the paper; materials, analysis tools or data.

## Data availability statement

Data included in article/supplementary material/referenced in article.

## Funding statement

This work was supported by the Institute for Information and Communications Technology Planning and Evaluation (IITP) grant funded by the Korean Government (MSIT) (No. 2022-0-01200, Training Key Talents in Industrial Convergence Security).

## Declaration of competing interest

The authors declare that they have no known competing financial interests or personal relationships that could have appeared to influence the work reported in this paper.
